# Supplementary material for: CRISPR/Cas9-mediated deletion of the Wiskott-Aldrich syndrome locus causes actin cytoskeleton disorganization in murine erythroleukemia cells
Source: PeerJ. 2019 Jan 16;7:e6284. doi: 10.7717/peerj.6284 (PMC6339507; doi:10.7717/peerj.6284)
Supplement: Table S1 [file peerj-07-6284-s001.docx]

|  | |
| --- | --- |
| **sgRNA** | **Sequences** |
| sgRNA WAS 1 | 5' CACCGAAGGCATATTGAGCCGGGCG 3' |
| sgRNA WAS 1 rc | 5' AAACCGCCCGGCTCAATATGCCTTC 3' |
| sgRNA WAS 2 | 5' CACCGTAAAGAAATACAGGCGACG 3' |
| sgRNA WAS 2 rc | 5' AAACCGTCGCCTGTATTTCTTTAC 3' |

**Suppl.Fig.1.** *sgRNA sequences for cloning into pX330 vector*
