# Supplementary material for: CRISPR/Cas9-mediated deletion of the Wiskott-Aldrich syndrome locus causes actin cytoskeleton disorganization in murine erythroleukemia cells
Source: PeerJ. 2019 Jan 16;7:e6284. doi: 10.7717/peerj.6284 (PMC6339507; doi:10.7717/peerj.6284)
Supplement: Table S2 [file peerj-07-6284-s002.docx]

|  | | | |
| --- | --- | --- | --- |
| **Primer** | **Sequences** | | **Amplicon**  **size**  **(bp)** |
| **ND** | Fwd | 5’-TGTGACGGTCGACATTCAGA-3’ | 191 |
|  | Rv | 5’-TCAGATGCTTTCCCACCCAT-3’ |  |
| **D** | Fwd | 5’-TATCATTGGCAACGTCCAAA-3’ | 602 |
|  | Rv | 5’-GACATGGTGGCACTTGTCTG-3’ |  |

**Suppl.Fig.S3.** *List of primers used for select biallelic deletion clones by PCR*
