# Supplementary material for: CRISPR/Cas9-mediated deletion of the Wiskott-Aldrich syndrome locus causes actin cytoskeleton disorganization in murine erythroleukemia cells
Source: PeerJ. 2019 Jan 16;7:e6284. doi: 10.7717/peerj.6284 (PMC6339507; doi:10.7717/peerj.6284)
Supplement: Figure S1 — A) Expression vector pcDNA3.1-Was (6,992 bp) containing the coding region of Was (blue) used in MEL-R stable transfections. B) Detailed genetic map of each of the three vectors used for the Was deletion in MEL cells by CRISPR/Cas9 technology. pX330-Was 1 (8,487 bp) and pX330-Was 2 (8,486 bp) showing its most relevant elements: sgRNA sequence (blue) and the coding sequence of SpCas9 (pink). pEFBOS-GFP (5,331 bp), expresses GFP (green). [file peerj-07-6284-s003.pdf]

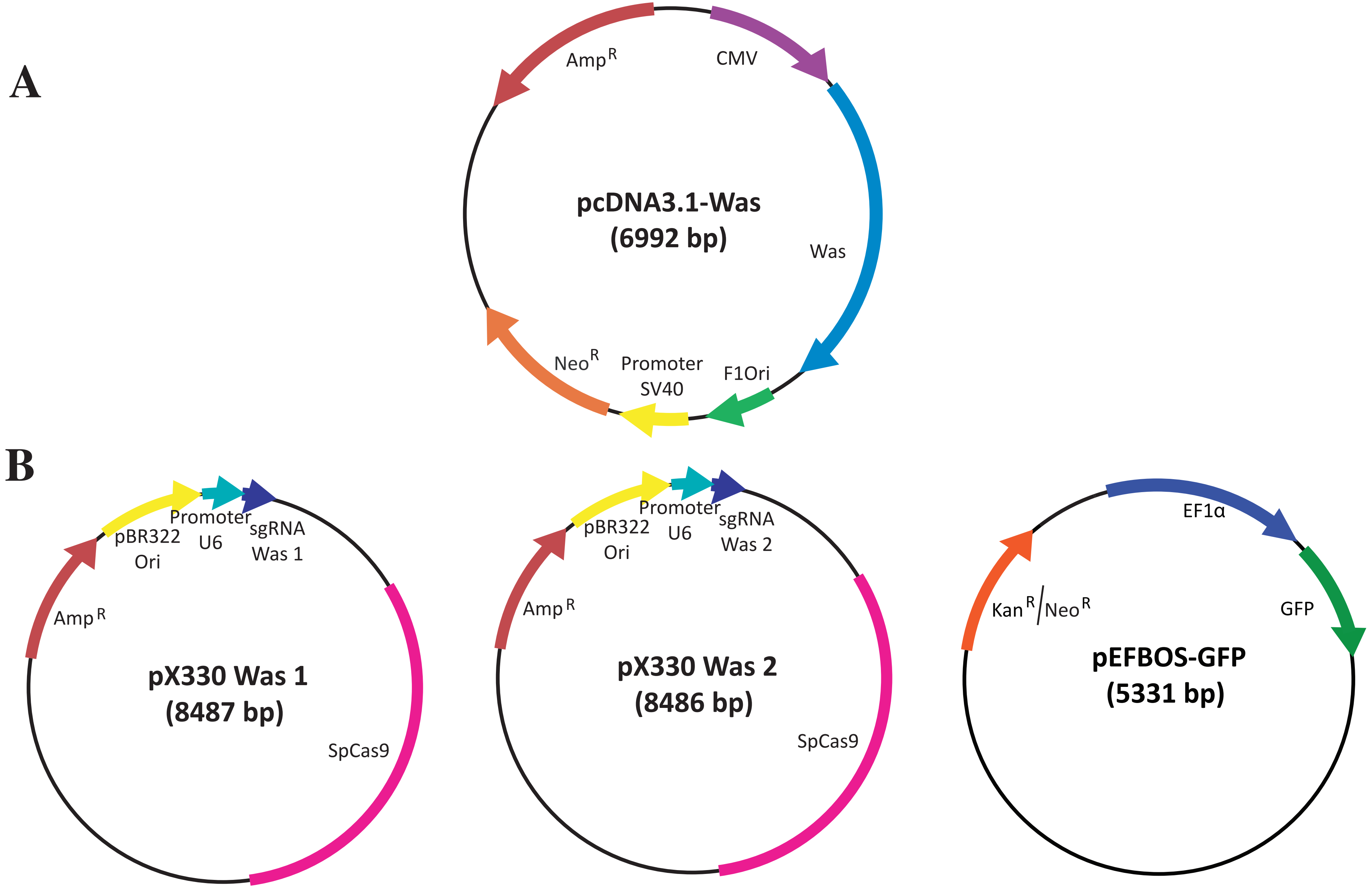

Suppl.Fig.S1. Maps of the vectors used in this study. A) Expression vector pcDNA3.1-Was (6,992 bp) containing the coding region of Was (blue) used in MEL-R stable transfections. B) Detailed genetic map of each of the three vectors used for the Was deletion in MEL cells by CRISPR/Cas9 technology. pX330-Was 1 (8,487 bp) and pX330-Was 2 (8,486 bp) showing its most relevant elements: sgRNA sequence (blue) and the coding sequence of SpCas9 (pink). pEFBOS-GFP (5,331 bp), expresses GFP (green).
