# Supplementary material for: CRISPR/Cas9-mediated deletion of the Wiskott-Aldrich syndrome locus causes actin cytoskeleton disorganization in murine erythroleukemia cells
Source: PeerJ. 2019 Jan 16;7:e6284. doi: 10.7717/peerj.6284 (PMC6339507; doi:10.7717/peerj.6284)
Supplement: Figure S2 — Western blot relative densitometric quantification using ImageJ corresponding to Figs. 1A and 1B (a and b), 3A and 3B (c and d), 6A and 6B (e and f) and 7 (g). Data are shown as the mean of three measurements. * P value < 0.05, ** P value < 0.01 [file peerj-07-6284-s004.pdf]

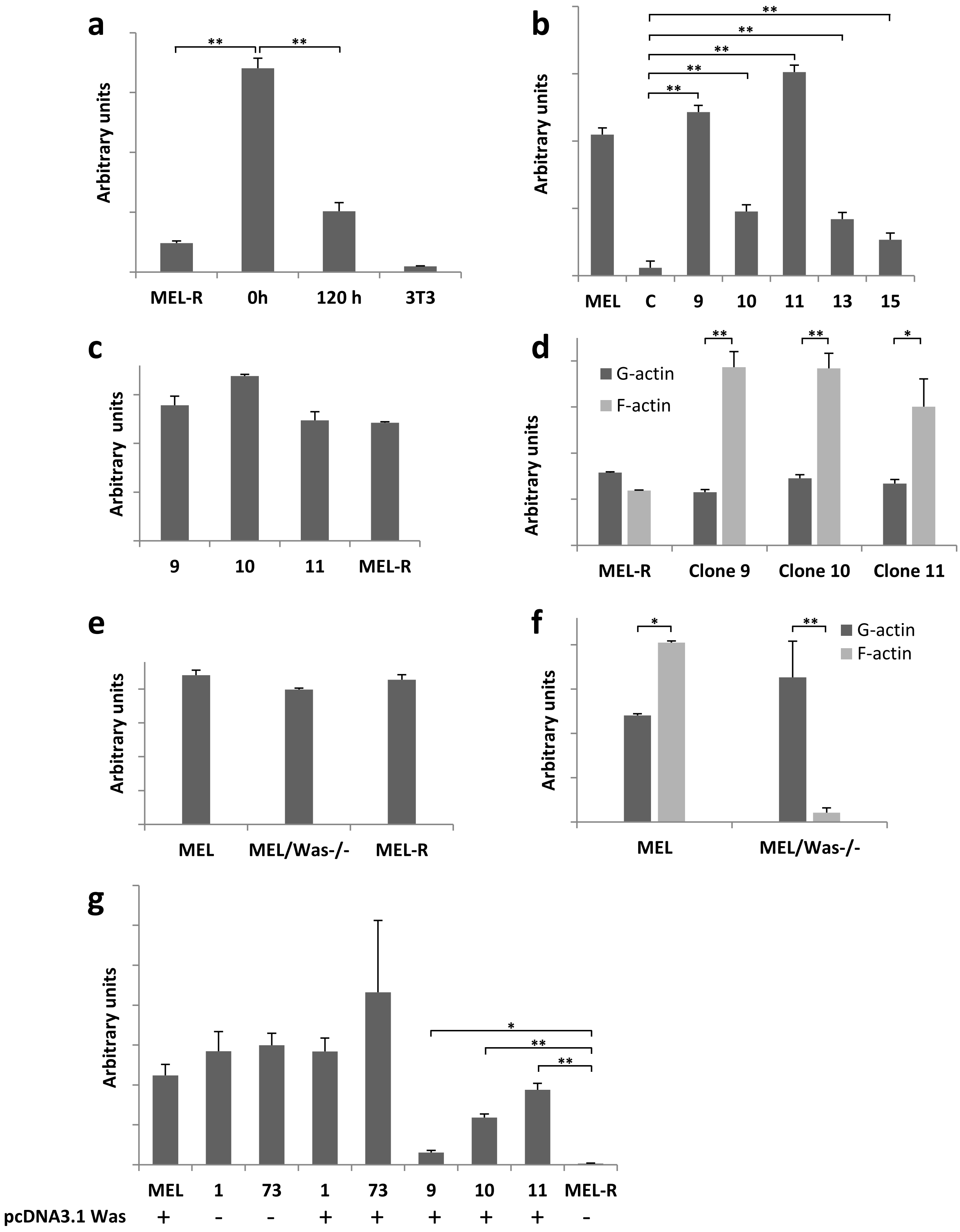

Suppl.Fig.S2. Western blot relative densitometric quantification using ImageJ corresponding to Figures 1A and B (a and b), 3A and B (c and d), 6A and B (e and f) and 7 (g). Data are shown as the mean of three measurements ± standard deviation. * P-value < 0.05, ** P-value < 0.01.
