# Supplementary material for: CRISPR/Cas9-mediated deletion of the Wiskott-Aldrich syndrome locus causes actin cytoskeleton disorganization in murine erythroleukemia cells
Source: PeerJ. 2019 Jan 16;7:e6284. doi: 10.7717/peerj.6284 (PMC6339507; doi:10.7717/peerj.6284)
Supplement: Figure S3 — Quantitative analysis of fluorescent images corresponding to Figs. 2 (a), 5 (b) and 8 (c). Normalized fluorescent intensity is expressed as arbitrary units (a.u.). Data are shown as the mean standard deviation (n < 40 cells per group). * P value < 0.05, ** P value < 0.01. [file peerj-07-6284-s005.pdf]

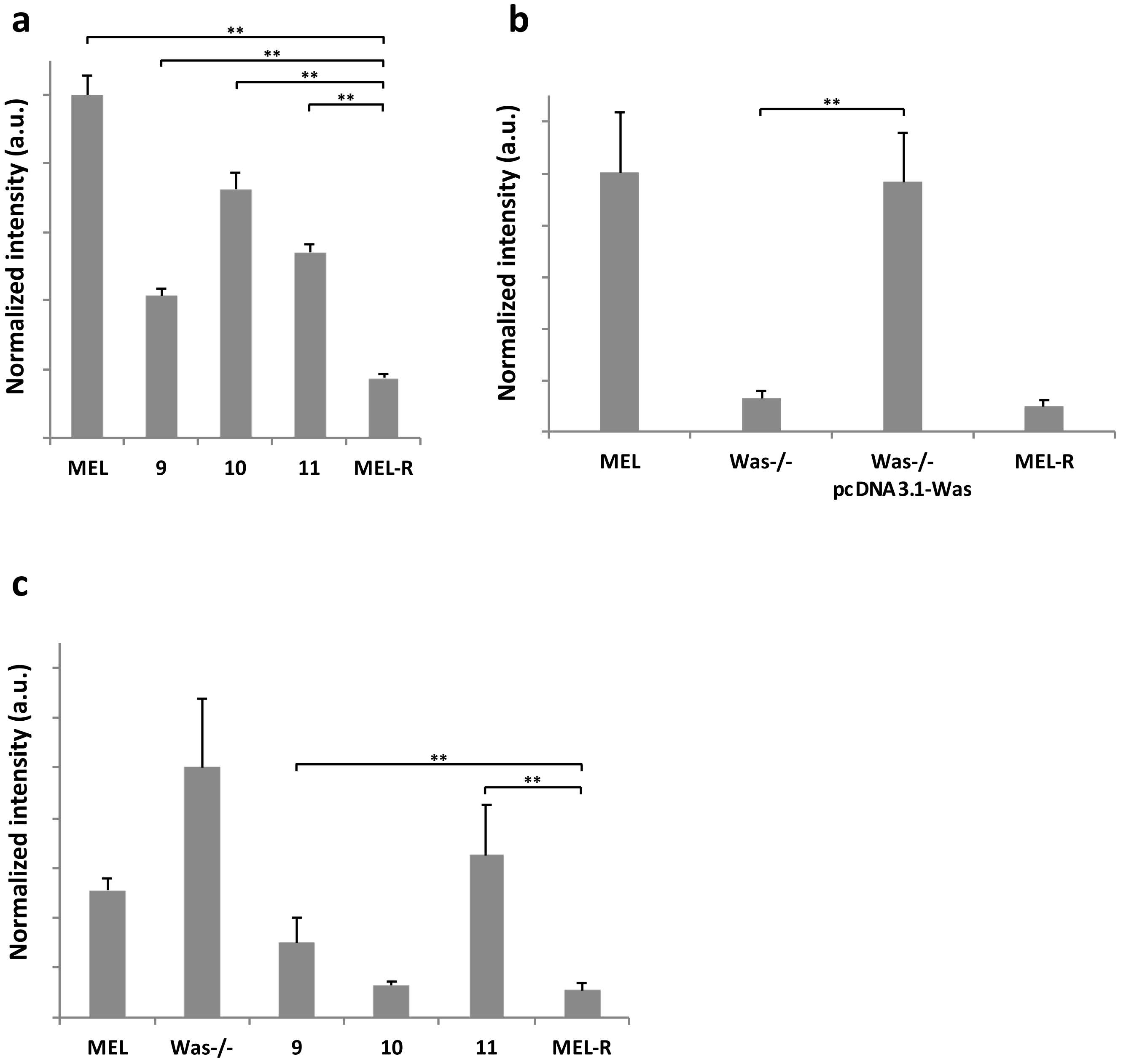

Suppl.Fig.S3. Quantitative analysis of fluorescent images corresponding to Figures 2 (a), 5 (b) and 8 (c). Normalized fluorescent intensity is expressed as arbitrary units (a.u.). Data are shown as the mean ± standard deviation ($n \geq 40$ cells per group). * P-value < 0.05, ** P-value < 0.01.
