# Supplementary material for: CRISPR/Cas9-mediated deletion of the Wiskott-Aldrich syndrome locus causes actin cytoskeleton disorganization in murine erythroleukemia cells
Source: PeerJ. 2019 Jan 16;7:e6284. doi: 10.7717/peerj.6284 (PMC6339507; doi:10.7717/peerj.6284)
Supplement: Figure S4 — (A) Growth kinetics of MEL and MEL/Was−∕ − cells measured every 24 hours over 4 days. (B) Percentage of differentiation in MEL cells and MEL/Was−∕ − transfectants. Differentiated cells (B+) were determined by benzidine assay. Cells were cultured in the presence of 5 mM HMBA for 96 h. Each bar in (A) and (B) indicates the standard deviation of three independent experiments. [file peerj-07-6284-s006.pdf]

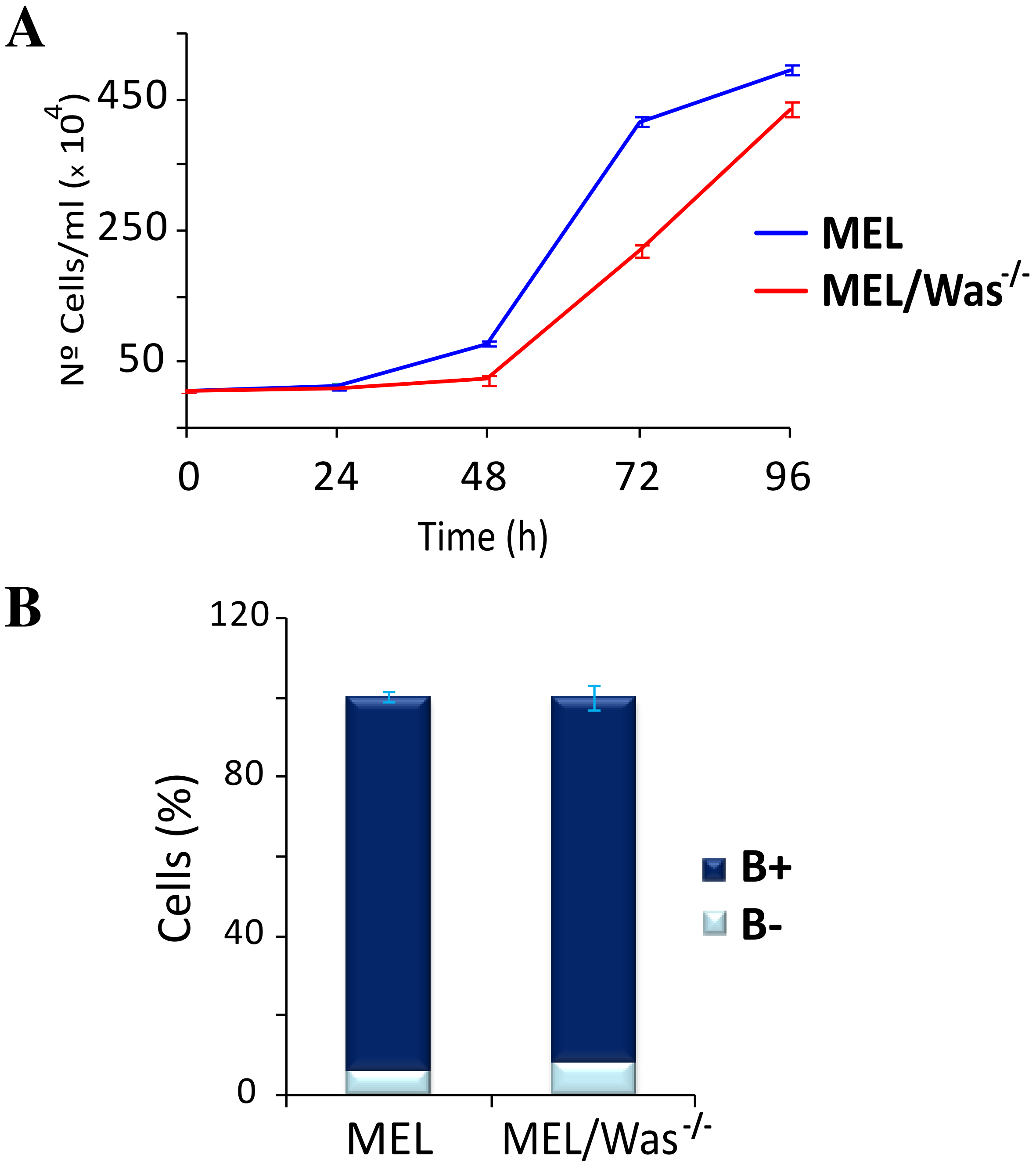

Suppl.Fig.S4. Growth kinetics and HMBA-mediated differentiation in MEL/Was -/- cells.
A) Growth kinetics of MEL and MEL/Was-/- cells measured every 24 hours over 4 days. B) Percentage of differentiation in MEL cells and MEL/Was-/- transfectants. Differentiated cells (B+) were determined by benzidine assay. Cells were cultured in the presence of 5 mM HMBA for 96 hours. Each bar in A) and B) indicates the standard deviation of three independent experiments.
