# Supplementary material for: CRISPR/Cas9-mediated deletion of the Wiskott-Aldrich syndrome locus causes actin cytoskeleton disorganization in murine erythroleukemia cells
Source: PeerJ. 2019 Jan 16;7:e6284. doi: 10.7717/peerj.6284 (PMC6339507; doi:10.7717/peerj.6284)
Supplement: Data S1 [file peerj-07-6284-s007.zip › Raw data legends.docx]

Raw data Fig.1A. Correspond to Figure 1A of the manuscript. Western blotting for Was for MEL-R (R), MEL undifferentiated cells (MEL), differentiated cells (120h HMBA) and fibroblasts (3T3).

Raw data Fig.1A’. Correspond to Figure 1A. Same membrane blotted for tubulin as a loading control.

Raw data Fig.1B. Correspond to Figure 1B. Western blotting for Was for stable transfectants (clones) overexpressing Was. MEL cells (MEL), MEL-R cells (R) and MEL-R transfected with an empty vector (Control 1 and 2) are also included.

Raw data Fig.1B’. Correspond to Figure 1B. Same membrane blotted for tubulin as a loading control.

Raw data Fig.3A. Correspond to Figure 3A (upper panel). Western blotting for β-actin for Was stable transfectants 9, 10 and 11.

Raw data Fig.3A’. Correspond to Figure 3A (low panel). Western blotting for α-tubulin as a loading control.

Raw data Fig.3B. Correspond to Figure 3B. Western blotting for β-actin in pellets and supernatants (G and F actin) of clones 9, 10 and 11. The 10’-lysis samples were used for Figure 3 in the manuscript.

Raw data Fig.3B’. Correspond to Figure 3B (upper panel). Western blotting for β-actin in pellets and supernatants (G and F actin) of MEL-R cells.

Raw data Fig. 4C. Correspond to Fig. 4C. MEL, MEL-R and three Was/KO-CRISPR (clones 1, 4 and 73) were blotted with Was antibody.

Raw data Fig. 4C’. Correspond to Fig.4C. Same as above, blotted with anti-tubulin as a loading control.

Raw data Fig. 6A. Correspond to Figure 6A. Samples from MEL, Was/KO (clones 73, 4 and 1) and MEL-R (R) were blotted with an anti-actin antibody.

Raw data Fig.6A’. Correspond to Figure 6A. Same as above, blotted with anti-tubulin as a loading control.

Raw data Fig.6B’. Correspond to Figure 6B (upper panel). Western blotting for β-actin in pellets and supernatants (G and F actin) of MEL cells.

Raw data Fig.6B. Correspond to Figure 6B (low panel). Western blotting for β-actin in pellets and supernatants (G and F actin) of Was/KO, clon1. Other data (Plek, Btk) are not included in this paper.

Raw data Fig.7. Correspond to Figure 7. Western blotting for Btk for MEL samples, CRISPR KO Was1 and Was73 (transfected with pcDNA 3.1-Was, first two), stable clones 9, 10 and 11 overexpressing Was and MEL-R cells.

Raw data Fig.7’. Correspond to Figure 7. Same as above, blotted with anti-tubulin as a loading control.
