# Supplementary figures and images for: CRISPR/Cas9-mediated deletion of the Wiskott-Aldrich syndrome locus causes actin cytoskeleton disorganization in murine erythroleukemia cells
Source: PeerJ. 2019 Jan 16;7:e6284. doi: 10.7717/peerj.6284 (PMC6339507; doi:10.7717/peerj.6284)

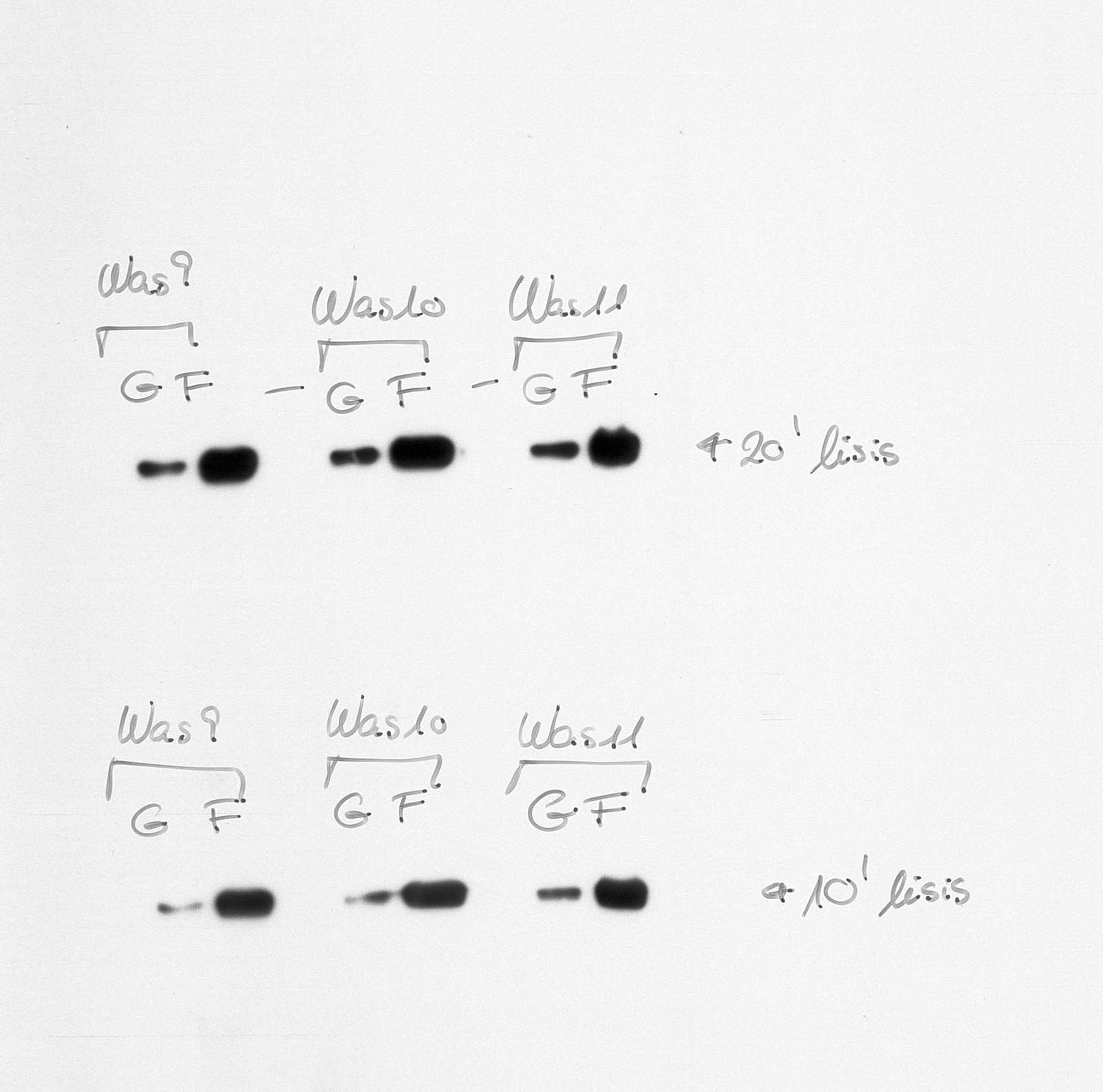

Supplement: Data S1 [file peerj-07-6284-s007.zip › Raw data Fig.3B.tif]

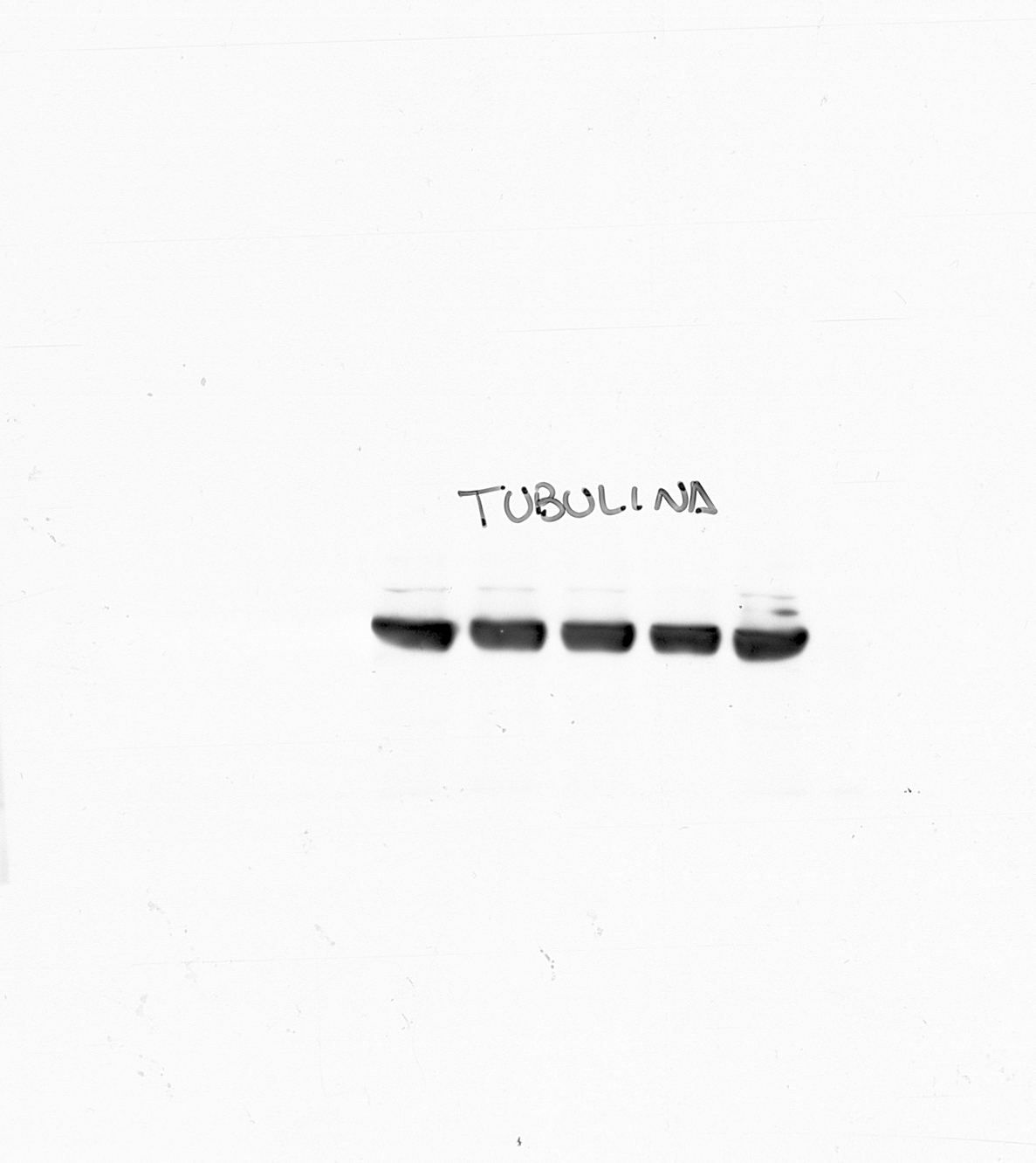

Supplement: Data S1 [file peerj-07-6284-s007.zip › Raw data Fig.4C'.tif]

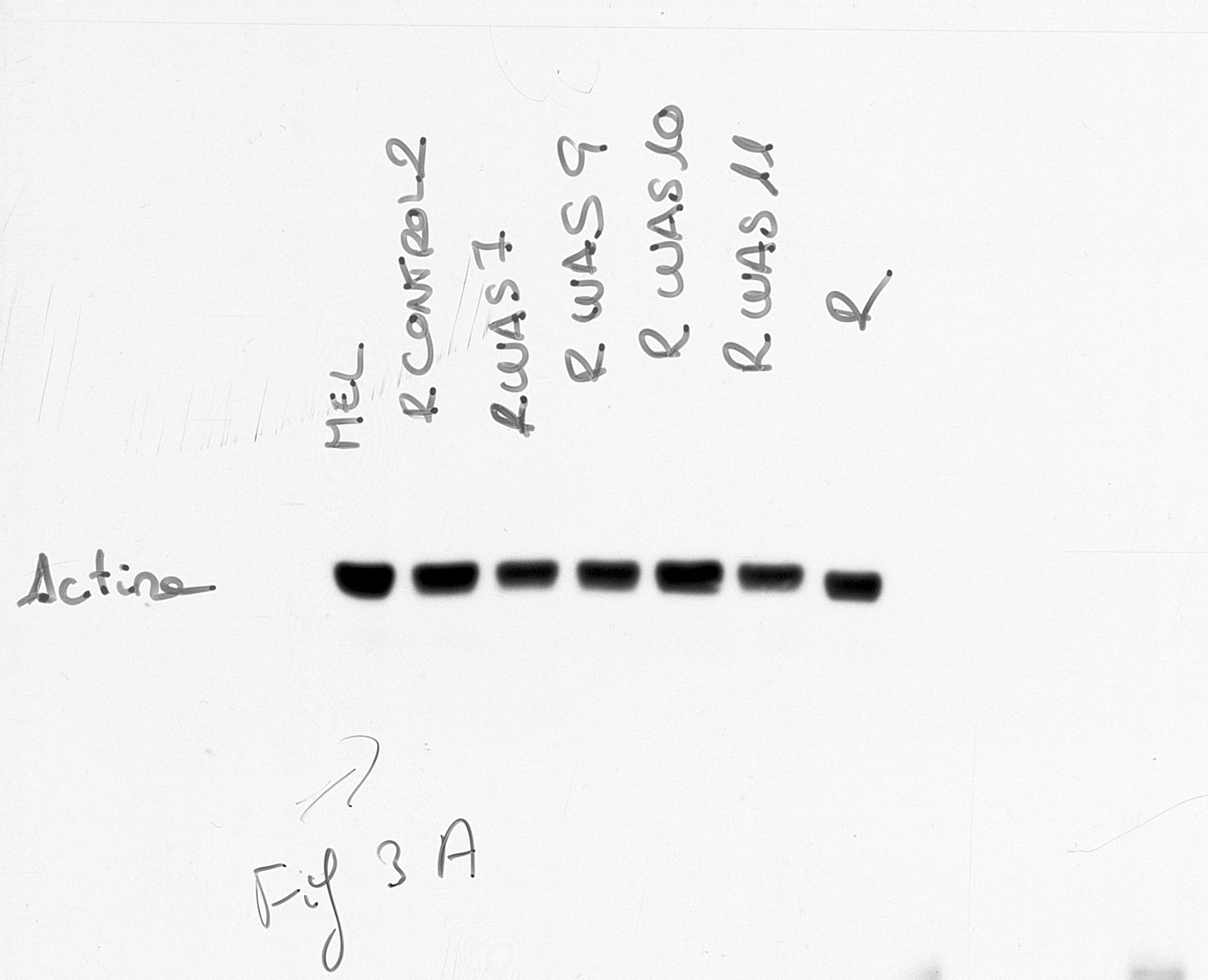

Supplement: Data S1 [file peerj-07-6284-s007.zip › Raw data Fig.3A.tif]

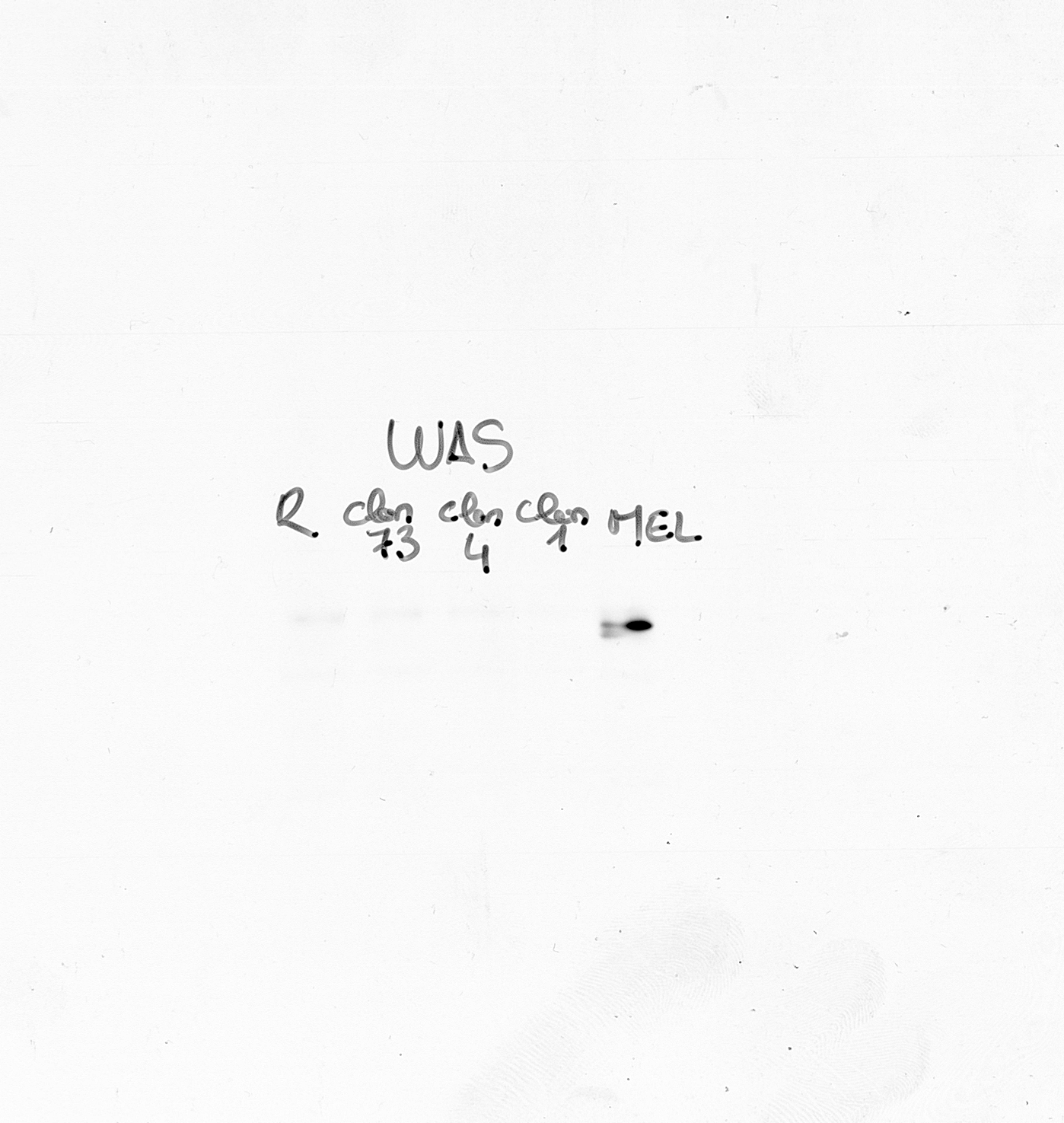

Supplement: Data S1 [file peerj-07-6284-s007.zip › Raw data Fig.4C.tif]

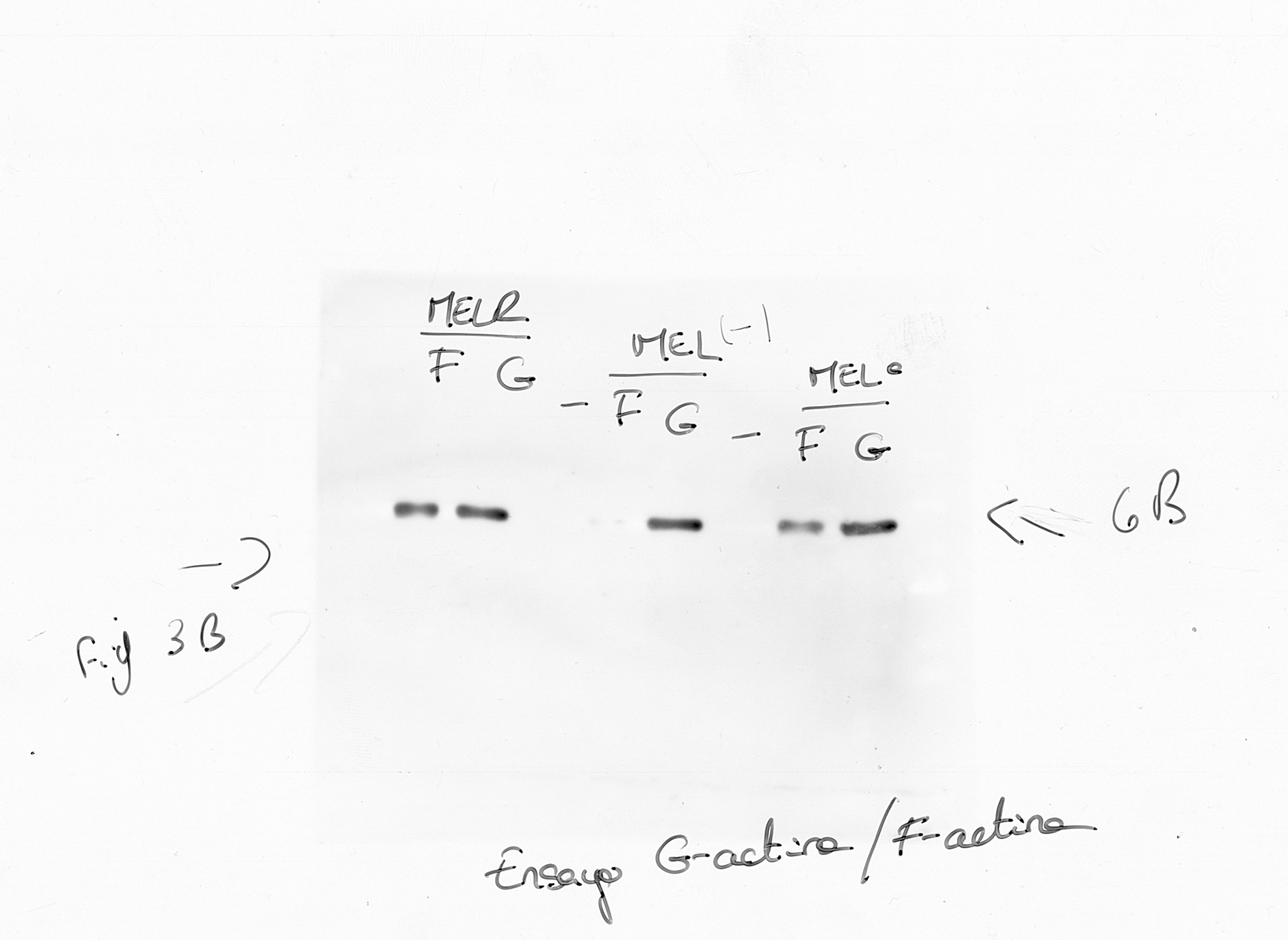

Supplement: Data S1 [file peerj-07-6284-s007.zip › Raw data Fig.3B'.tif]

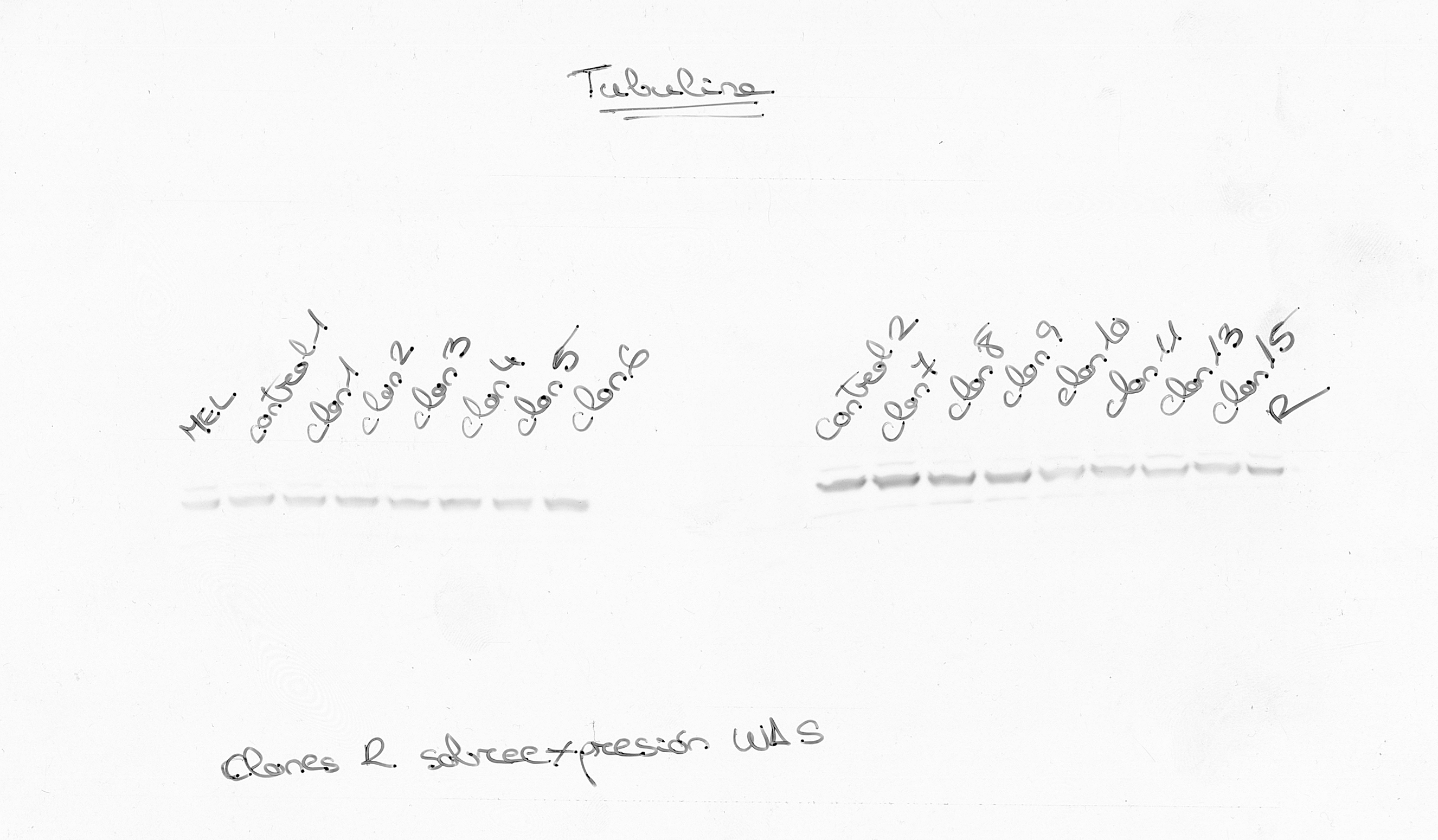

Supplement: Data S1 [file peerj-07-6284-s007.zip › Raw data Fig.1B'.tif]

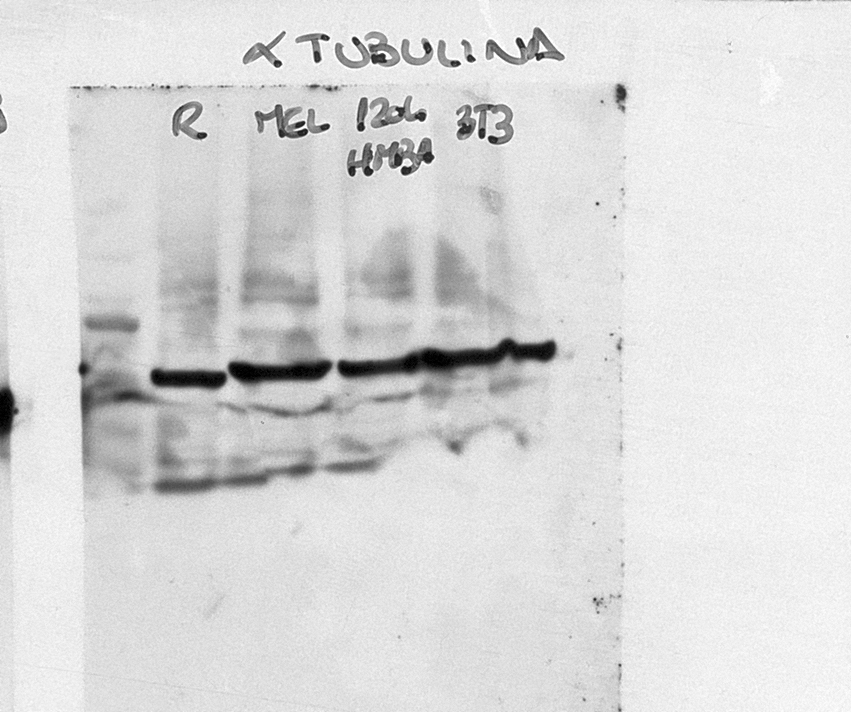

Supplement: Data S1 [file peerj-07-6284-s007.zip › Raw data Fig.1A'.tif]

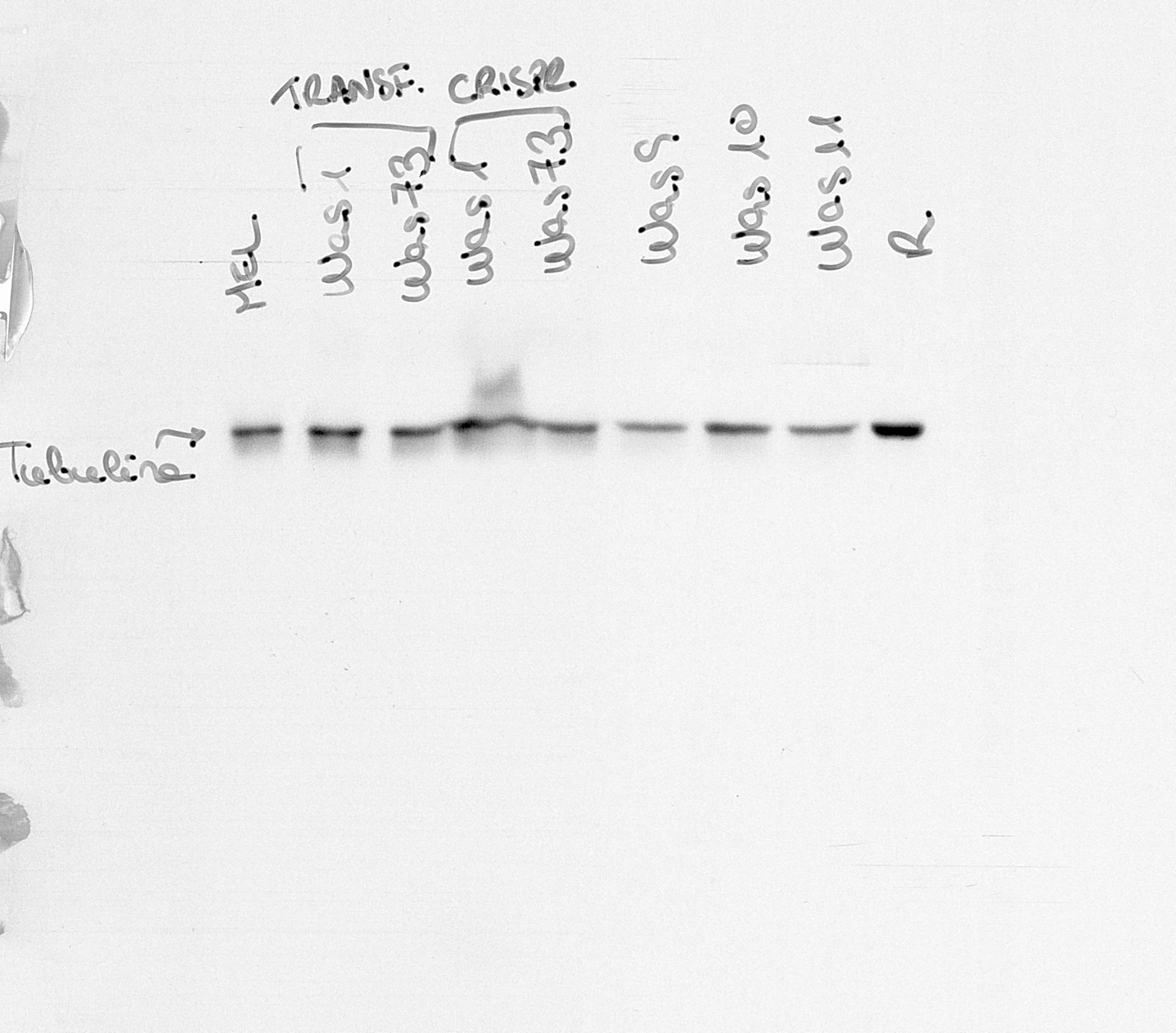

Supplement: Data S1 [file peerj-07-6284-s007.zip › Raw data Fig.7'.tif]

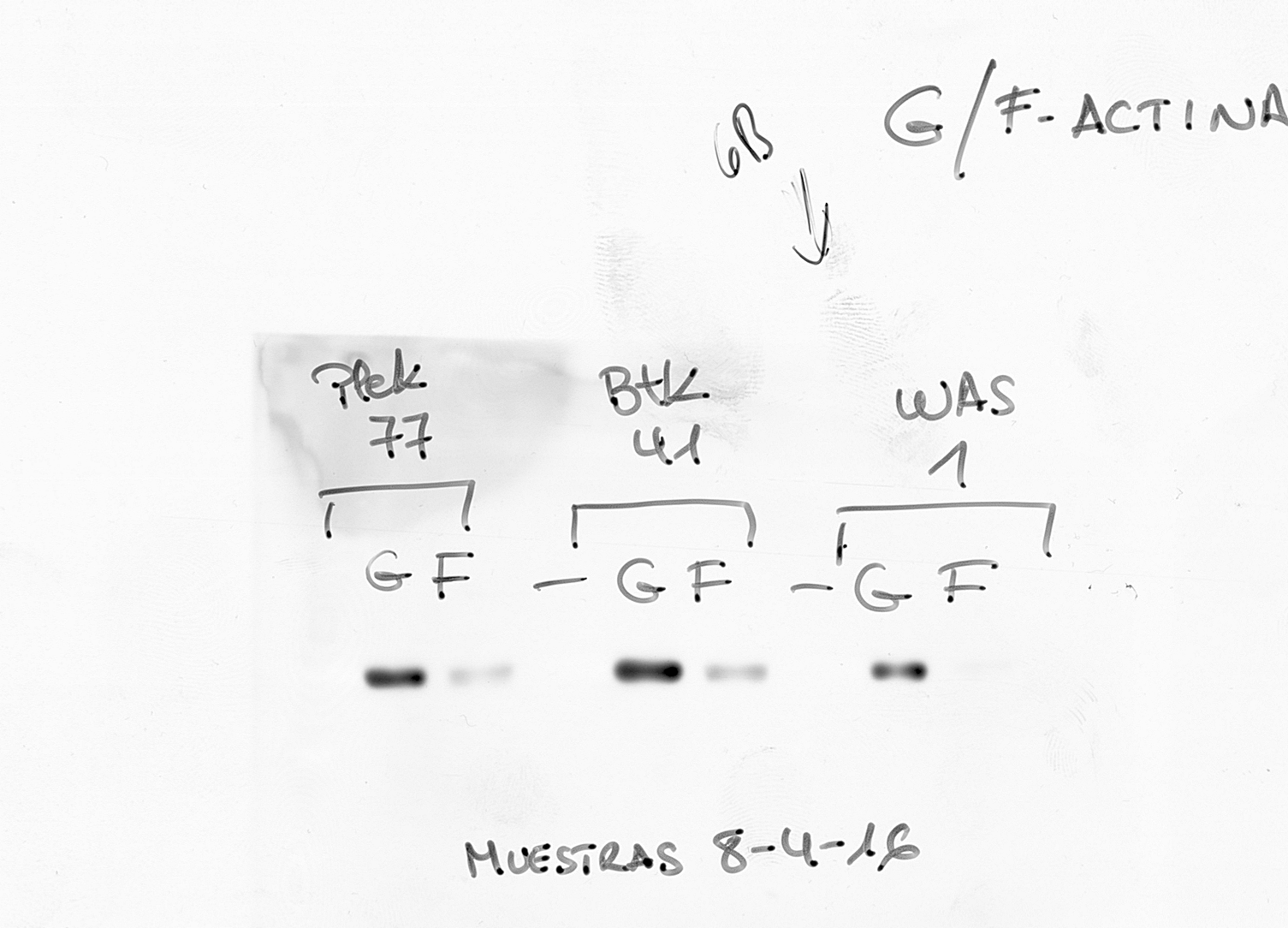

Supplement: Data S1 [file peerj-07-6284-s007.zip › Raw data Fig.6B.tif]

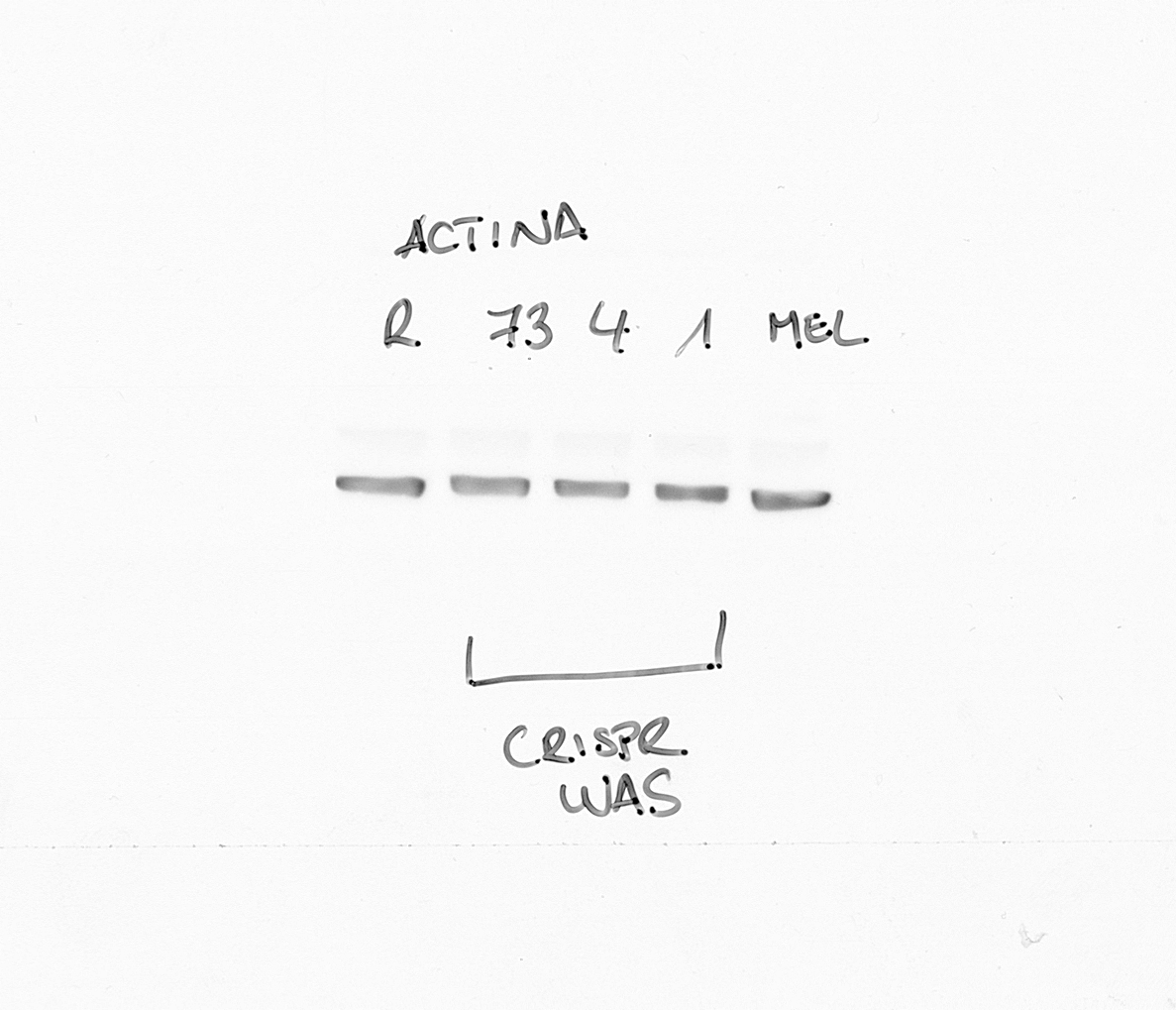

Supplement: Data S1 [file peerj-07-6284-s007.zip › Raw data Fig.6A.tif]

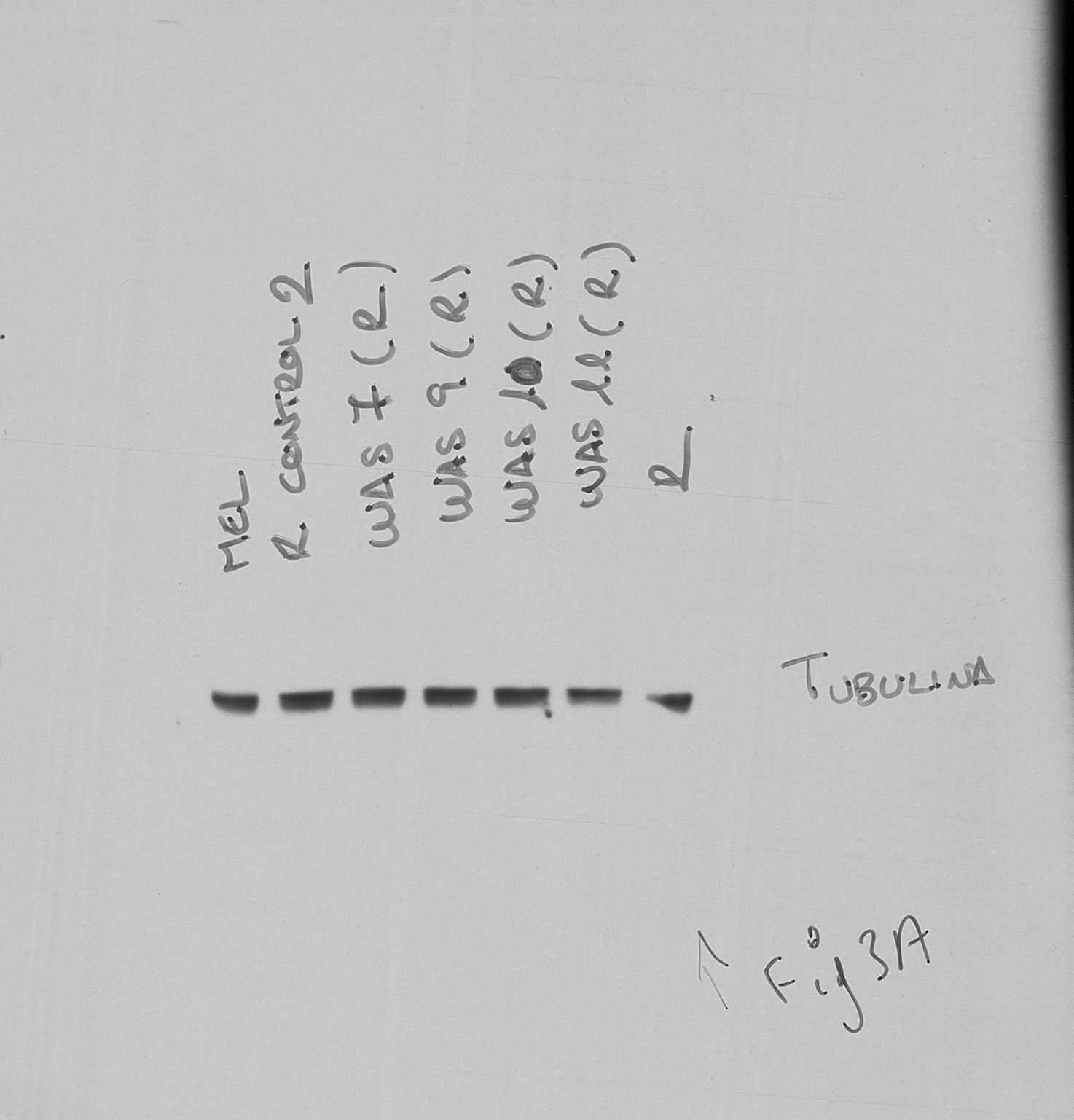

Supplement: Data S1 [file peerj-07-6284-s007.zip › Raw data Fig.3A'.tif]

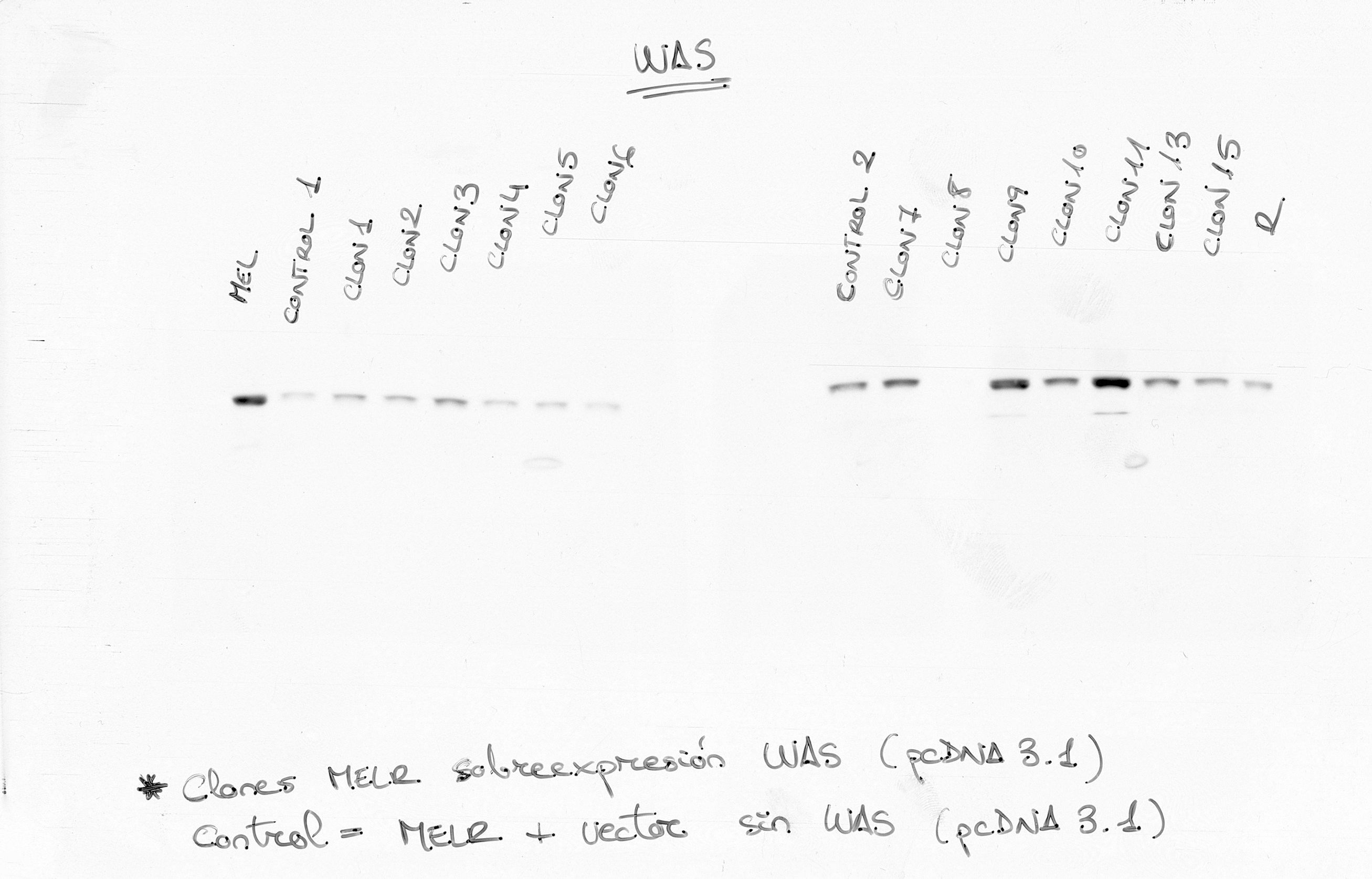

Supplement: Data S1 [file peerj-07-6284-s007.zip › Raw data Fig.1B.tif]

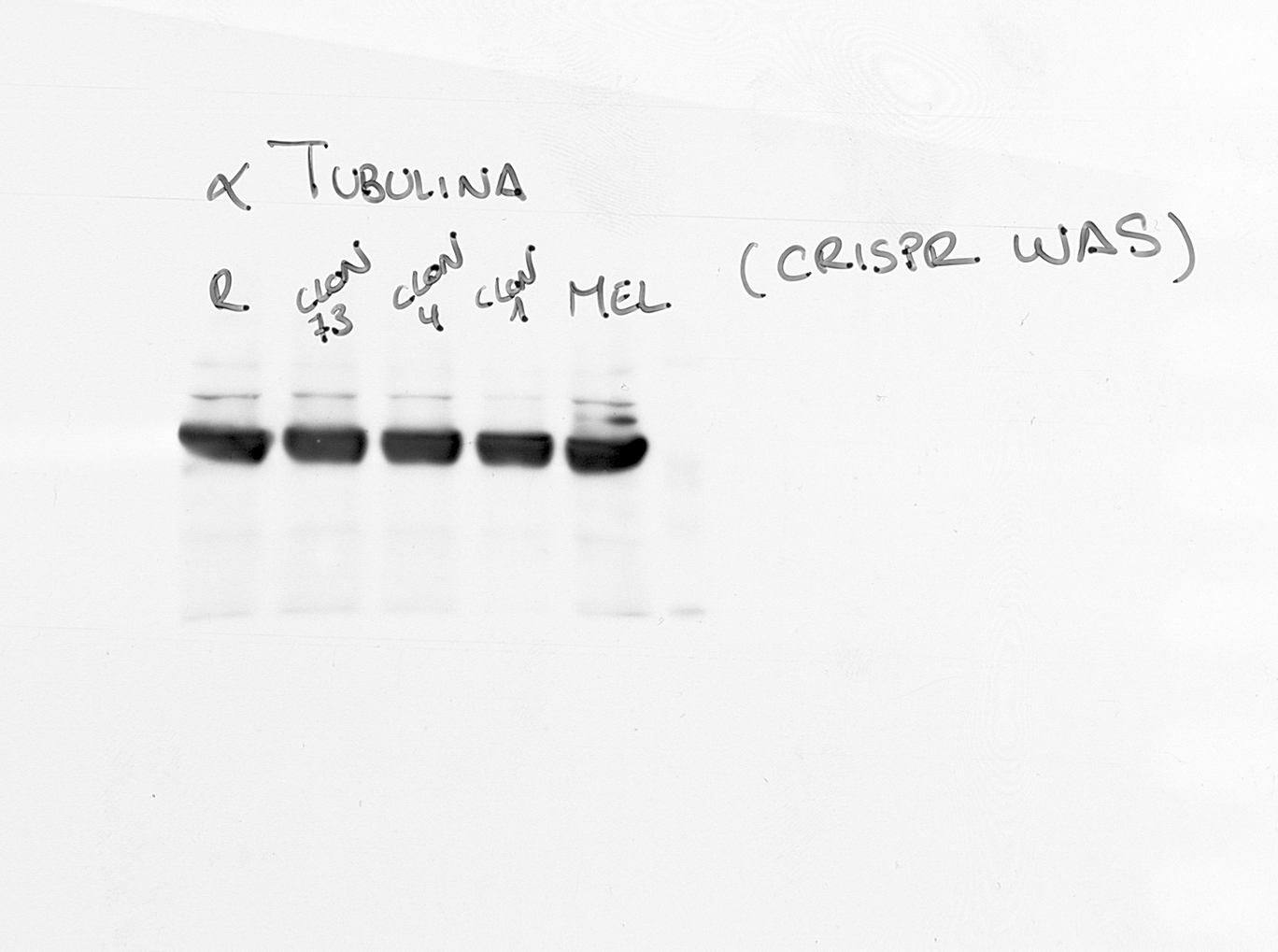

Supplement: Data S1 [file peerj-07-6284-s007.zip › Raw data Fig.6A'.tif]

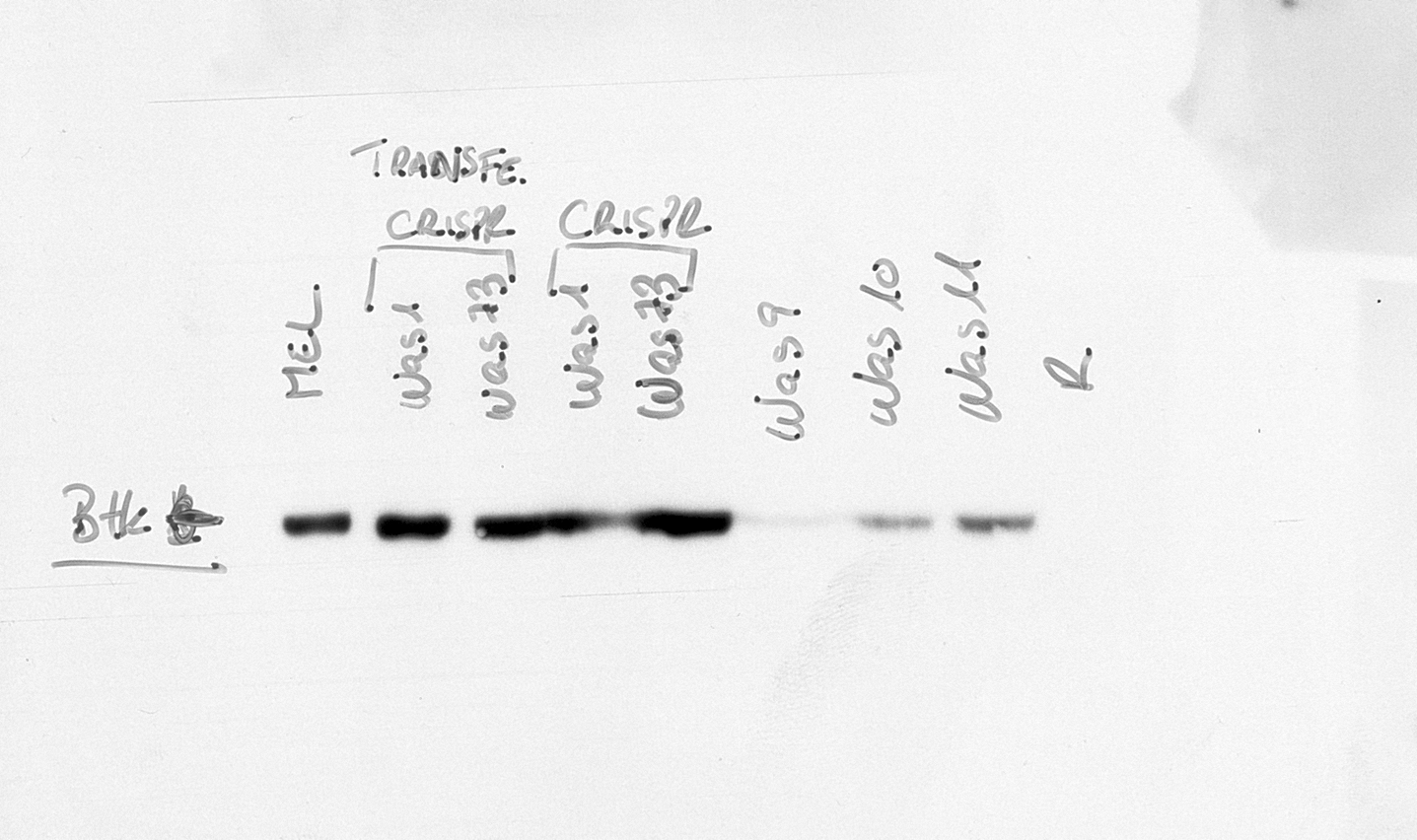

Supplement: Data S1 [file peerj-07-6284-s007.zip › Raw data Fig.7.tif]

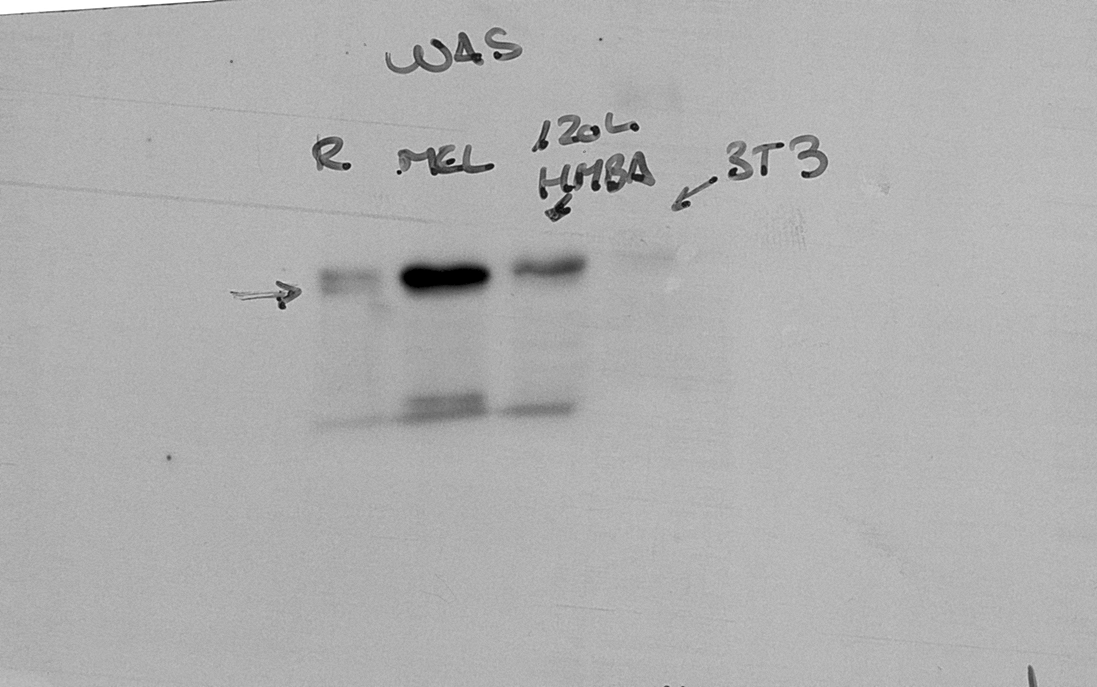

Supplement: Data S1 [file peerj-07-6284-s007.zip › Raw data Fig.1A.tif]
